# Influence of acute hyperlipidemia to adipocyte-derived hormones in lean normotensive and subjects with metabolic syndrome

**DOI:** 10.1186/1758-5996-6-132

**Published:** 2014-12-02

**Authors:** Heno F Lopes, Richard L Klein, W Timothy Garvey, Theodore Goodfriend, Brent M Egan

**Affiliations:** Department of Medicine, Medical University of South Carolina, Charleston, South Carolina USA; Department of Pharmacology, Medical University of South Carolina, Charleston, South Carolina USA; Unidade de Hipertensao-Heart Institute (InCor), University of Sao Paulo Medical School, Av Dr Enéas C. de Aguiar, 44, Zip code 05403-904 São Paulo, Brazil; Universidade Nove de Julho - UNINOVE, University of São Paulo Medical School, Av Dr Enéas C. de Aguiar, 44, Zip code 05403-904 São Paulo, Brazil; Department of Medicine and Pharmacology, William S. Middleton Veterans Hospital University of Wisconsin, Madison, Wisconsin USA

**Keywords:** Intralipid^®^ and heparin, Adiponectin, Leptin, ASP/C3adesARG, Lean normotensives, Metabolic syndrome subjects

## Abstract

**Background:**

Adipocyte-derived factors and regulators likely contribute to the metabolic syndrome (MetS) in patients with central obesity. This study was undertaken to assess the contribution of leptin, adiponectin, and acylation stimulating protein (ASP-C3ades/ARG) to hemodynamic (blood pressure [BP]) and metabolic (insulin, glucose, lipids) features of MetS.

**Methods:**

In this study, leptin, adiponectin, and C3ades/ARG were measured at baseline and in response to an infusion of Intralipid^®^ and heparin in 12 lean healthy controls and 12 patients with MetS.

**Results:**

Baseline plasma leptin (27.6 ± 6.2 vs. 10.9 ± 3.8 ng/mL, p < 0.01) and plasma C3ades/ARG (273 ± 79 vs 198 ± 57 mg/dL, p < 0.05) were higher in the MetS than control group, whereas baseline plasma adiponectin was higher in the control than MetS group (9.9 ± 1.9 vs. 5.4 ± 0.6 g/mL). Plasma leptin correlated with body mass index (BMI), systolic and diastolic BP (r = 0.53-0.77, p < 0.01). Conversely, adiponectin correlated inversely with insulin, glucose, waist circumference, and insulin sensitivity (r = 0.48-0.51, p ≤ 0.02). Plasma triglycerides increased similarly in MetS and control groups after 4-hours of Intralipid and heparin. C3ades/ARG increased only in lean volunteers. The decrease in triglycerides 1-hour post-infusion was lower in the MetS than control group (-116 ± 33 vs. -282 ± 81 mg/dL, p = 0.01) and correlated inversely with the change in C3ades/ARG.

**Conclusion:**

These data suggest that leptin is more closely associated with hemodynamic (BP) aspects of MetS, whereas adiponectin and C3ades/ARG are more closely associated with metabolic components.

## Background

A rapidly growing body of literature indicates that adipocyte-derived signaling hormones and regulators of adipocyte function play an important role in the pathogenesis and complications of metabolic syndrome (MetS). Leptin, an adipocyte secreted hormone, has multiple cardiovascular functions and is associated with sympathetic activation, angiogenesis, oxidative stress and thrombosis [1–4]. Adiponectin, a novel adipocyte-derived collagen-like protein, has been associated linked to anti-inflammatory and anti-atherogenic proprieties [5, 6]. Leptin and adiponectin were inversely related in normal controls and obese women [7]. C3adesARG (ASP) is a complement C3a component resulted from the removal of the carboxy-terminal arginine. ASP/C3adesARG increases postprandially and correlated with the rate of triglyceride clearance in one study, but levels did not change post-prandially in another report [8, 9]. ASP/C3adesARG is an adipocyte product that correlated with postprandial lipemia in subjects with coronary artery disease [10]. These observations suggest that different adipocyte-derived factors and the balance between these factors contribute to various features of MetS and its cardiovascular diseases risk factors. At least some of these factors are responsive to an acute oral and/or parenteral lipid load. The aim of this study was to evaluate the relationship of plasma leptin, adiponectin, and ASP/C3adesARG to various aspects of MetS and the response of these factors to acute hyperlipidemia. Subjects for this study included obese subjects with features of the MetS and age, gender, and race-matched lean, healthy volunteers.

## Methods

### Subjects

Twelve subjects with features of MetS (waist circumference ≥88 cm for women and ≥102 cm for men, triglycerides >150 mg/dL and/or HDL-cholesterol <45 for women or <40 mg/dL for men, BP <130-159/85-99 mmHg) and 12 healthy volunteers (waist circumference <88 cm for women and <102 cm for men, BP <130/85 mmHg) with normal lipids matched for age, race, and gender were evaluated as described previously [11]. In brief, subjects read and signed a written informed consent document approved by the Institutional Review Board of Medical University of South Carolina. They were scheduled for 3 consecutive weekly screening visits to ensure eligibility. Subjects were considered in MetS group when they met at least 3 criteria defined by the Adult Treatment Panel III classification [12].

### Physiological data

During the qualifying visits, subjects had their blood pressure and heart rate measured in triplicate in the sitting position. Blood pressures were measured by a trained observer using a mercury sphygmomanometer and appropriately sized cuff. The first Korotkoff sound defined systolic blood pressure and the disappearance of 5th Korotkoff sound determined diastolic blood pressure. Heart rate was measured by palpation of the radial pulse for 30 seconds between the 2nd and 3^rd^ measurements of blood pressure with subjects in the seated position.

### Biochemical measurements

Blood for non-esterified fatty acids (NEFAs) was drawn into prechilled Eppendorf tubes containing paraoxon [13]. Plasma was stored at -70°C before analyzing the total plasma NEFAs using the 63Ni method [14].

#### Lipids and lipoproteins

Triglycerides were measured by fluorometric method. Total cholesterol was measured by the colorimetric method, and HDL- cholesterol was prepared from whole plasma by precipitation with phosphotungstate-MgCl2 [15]. LDL-cholesterol and VLDL-cholesterol were calculated [16].

#### Adipocyte factors

Plasma leptin was measured by radioimmunoassay method and plasma adiponectin was measured by the Elisa method. Plasma ASP/C3adesARG was measured by the Elisa sandwich Immunoassay method. Total antioxidant capacity was measured by the ferric reducing activity of plasma (FRAP) assay under fasting conditions.

#### Total antioxidant capacity

FRAP assay was performed as previously described [17]. FRAP activity was measured in triplicate on each sample and the mean of the three values was used in the analysis.

#### Insulin and HOMAir

Plasma insulin was measured by radioimmunoassay method and the homeostatic model assessment for insulin sensitivity (HOMAir) was calculated dividing glucose (mmol/L) × insulin (IU/L) by 22.5.

### Protocol

On the study day subjects were admitted to the outpatient General Clinical Research Center (GCRC) at 08:00 following an overnight fast. Intravenous access was established in both arms, with one side for obtaining blood samples and the other for infusion of Intralipid^®^ and heparin. After 30 minutes of resting blood samples were drawn for serum lipids and plasma glucose, insulin, total anti-oxidant capacity (FRAP assay), NEFAs, triglycerides, adiponectin, and ASP/C3adesARG. An infusion was then started of 20% Intralipid^®^ (Baxter Healthcare Corp., Glendale, CA) at 0.8 ml/m2/min and heparin (200 U bolus, followed by infusion at 1000 U/h). Heparin was given to activate lipoprotein lipase and accelerate the hydrolysis of fatty acids from triglycerides. Blood samples were obtained for triglycerides, NEFAs, leptin, adiponectin, insulin, and ASP/C3adesARG at 2-hours and 4-hours of the Intralipid^®^ and heparin infusion.

### Statistical analysis

Group differences in dichotomous variables including gender and race were examined using the Chi-square test. Age, casual blood pressure and heart rate were shown as median, minimum and maximum and body mass index (BMI) in media and standard error. The Student’s unpaired t-test was used to compare casual blood pressure, heart rate and BMI. Changes in variables over time during the infusion of Intralipid^®^ and heparin were assessed using the General Linear Model for repeated measures. Correlations were determined by Pearson’s method. Statistical analyses were performed with the SPSS/PC 20.0 statistical software package (SPSS, Chicago, IL). A p-value <0.05 was accepted as statistically significant. All multiple comparisons procedures were taken into consideration after False Discovery Rate (FDR) as described by Benjamini and Hochberg [18].

## Results and discussion

**Figure 1:**
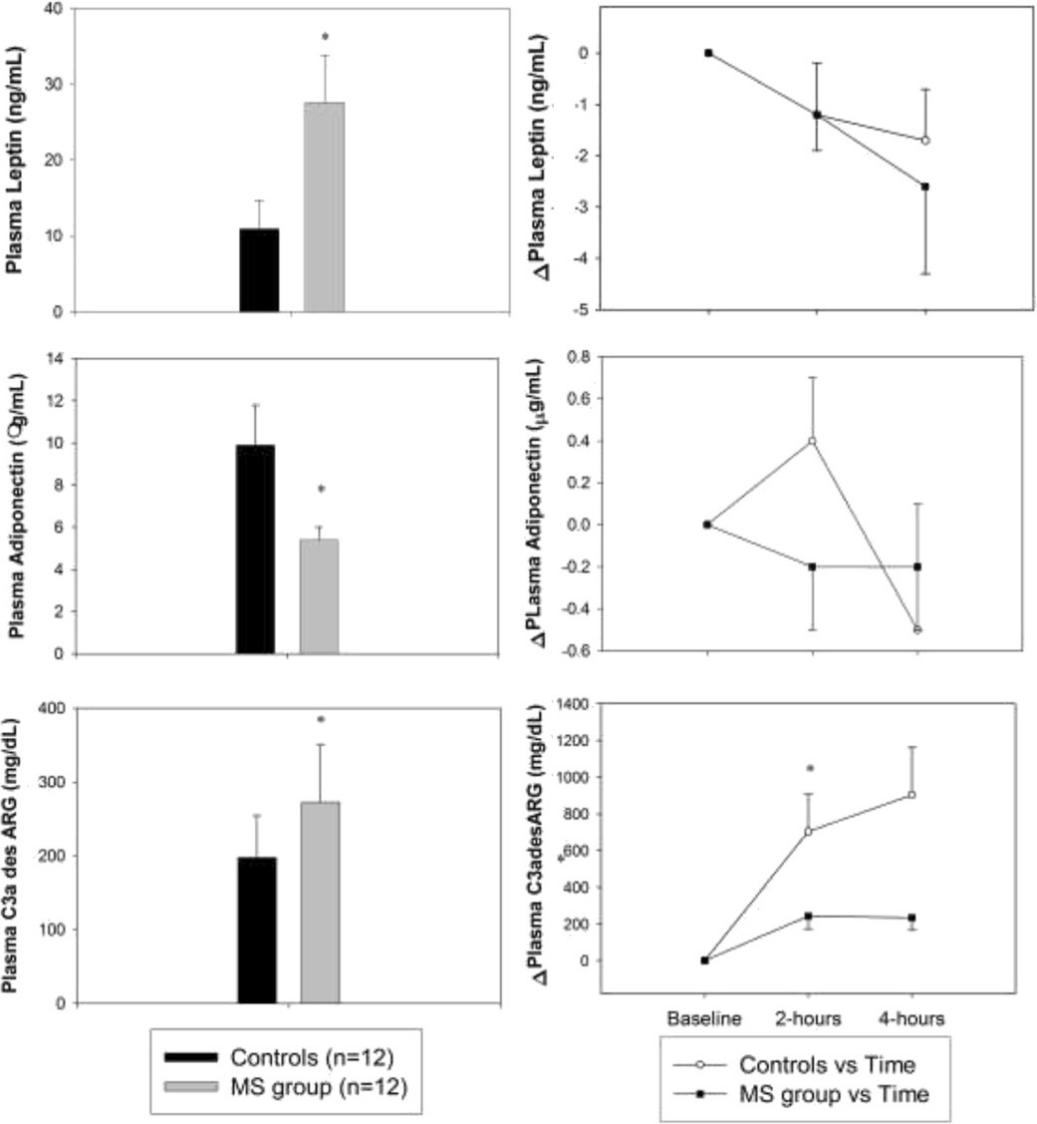
**Plasma leptin, adiponectin and ASP/C3adesARG values at baseline and after Intralipid** ^**®**^ **infusion in control and metabolic syndrome groups.** *p<0.05.

Demographic data for control and metabolic syndrome groups are shown in Table 1. Values for leptin, adiponectin, and ASP/C3adesARG at baseline and during the Intralipid^®^ and heparin infusion in MetS and control groups are shown in (Figure 1). As shown, baseline leptin and ASP/C3adesARG was higher and adiponectin lower in the MetS than the control group (Figure 1). Plasma leptin and adiponectin did not change during Intralipid and heparin infusion in both groups. However, plasma ASP/C3adesARG increased significantly in the control group after 2-hours of Intralipid^®^ and heparin infusion. The control group also showed a positive correlation between the changes in ASP/C3adesARG and triglycerides after 2-hours of Intralipid^®^ and heparin infusion (r = 0.80, p = 0.002). The MetS group showed a negative correlation between the changes for triglycerides and insulin during the Intralipid^®^ and heparin infusion (r = 0.80, p = 0.002; r = -0.65, p = 0.02 for 2 and 4-hours respectively).

**Table 1:** **Demographic and biochemistry data from subjects with MS and controls**

Variables	MS Group (n = 12)	Controls (n = 12)	P-value
Age (years)	35	38	NS
	28-46	26-47	
Gender (f/m)	6/6	6/6	NS
Race	7 AA, 5 C	6 AA, 6 C	NS
Systolic BP (mm Hg)	132	111	<0.0001
	(130-144)	(100-126)	
Diastolic BP (mm Hg)	91	74	<0.0001
	(86-94)	(64-82)	
Heart Rate (bpm)	77	71	<0.005
	(72-84)	(60-80)	
BMI (kg/m^2^)	34.1 ± 1.9	22.7 ± 0.5	<0.0001
Waist Circumference (cm)	106 ± 4	78 ± 3	<0.0001
Total-cholesterol (mg/dL)	229 ± 6	173 ± 7	<0.0001
HDL-cholesterol (mg/dL)	47 ± 3	61 ± 5	<0.05
LDL-cholesterol (mg/dL)	152 ± 5	95 ± 6	<0.0001
VLDL-cholesterol (mg/dL)	29 ± 4	16 ± 1	<0.01
Triglycerides (mg/dL)	171 ± 30	81 ± 6	0.01
Glucose (mg/dL)	90 ± 3	83 ± 3	0.08
Insulin (UI/L)	12.5 ± 1.	6.5 ± 0.9	0.01
Sodium (mmol/L)	139.3 ± 0.7	142.0 ± 0.7	0.01
Potassium (mmol/L)	4.34 ± 0.15	4.32 ± 0.08	NS

Data presented as median, minimum, maximum and mean ± SEM.

The correlations in all subjects combined between plasma leptin and body mass index (r = 0.77, p = 0.001), systolic BP (r = 0.73, p = 0.001), and diastolic BP (r = 57, p = 0.030) were significant. The correlation between leptin and waist circumference, and heart rate did not achieve significance after FDR correction. Adiponectin correlated inversely with waist circumference (r = 0.63, p = 0.008). Adiponectin correlated negatively with insulin, glucose, HOMAir (r = 0.50-0.63, p < 0.02) and positively with the total antioxidant capacity (r = 0.50, P < 0.02). We found a positive correlation between plasma ASP/C3adesARG and BMI, insulin, HOMAir, and heart rate. After FDR correction these correlations did not achieve significance.

Plasma NEFAs and triglyderides increased similarly in MetS and control groups after 4-hours of Intralipid^®^ and heparin infusion. However, the decrease in triglycerides 1-hour post-infusion was significantly lower in the MetS group compared to the control group (116 ± 33 versus 282 ± 81 mg/dL, p = 0.01).

In this study, subjects with MetS had higher plasma leptin, ASP/C3adesARG and lower adiponectin then the normal subjects (Figure 1).

These three adipocyte-derived factors showed a differential relationship to various components of MetS as described above. The findings are consistent with previous studies, which suggest that adipocytes play an important role in the pathogenesis of MetS and its complications. Collectively, evidence indicates that the level of and balance between specific adipocyte-derived peptides and lipids may modulate the expression of the hemodynamic and metabolic components of the insulin resistance syndrome.

Leptin has been implicated as a key transducing factor between obesity and hypertension [19]. Leptin’s pressor effects appear to be mediated in large part by activation of the sympathetic nervous system. While insulin and leptin are both elevated with obesity and both activate the nervous system, the actions of leptin appear to predominate [20, 21]. In this study, we found a positive correlation between plasma leptin and body mass index, systolic BP, and diastolic BP. These findings are consonant with those of Leyva, et al, who showed a positive correlation between leptin and BMI, systolic and diastolic BP [22]. Another important results in this study are related to the negative correlation between adiponectin insulin, glucose, HOMAir and waist circumference. These results suggest a negative correlation between insulin resistance and adiponectin. Since adiponectin, is an adipocyte-derived collagen-like protein that has been associated to anti-inflammatory and anti-atherogenic proprieties, it makes sense that subjects with features of insulin resistance, a cardiovascular risk factor, are supposed to show lower plasma adiponectin levels. We also found a positive correlation between adiponectin and the total antioxidant capacity (FRAP assay), although it disappeared after FDR correction. It suggests that the highest the total antioxidant capacity better are the anti-atherogenic and anti-inflammatory profiles.

Maslowska and coworkers showed a higher plasma ASP/C3adesARG in obese than non-obese subjects and suggested that ASP/C3adesARG pathway has involvement in the pathogenesis of obesity [23]. Since subjects with the features of MetS have a high triglycerides level comparing to normal subjects and the ASP/C3adesARG is associated to triglycerides clearance, it suggest that the higher plasma level of ASP/C3adesARG in obese subjects can be a sign of a malfunctioning mechanism of this protein. Plasma ASP/C3adesARG increased significantly in control group during Intralipid^®^ and heparin infusion. Meijssen et al showed an increased post-prandial ASP/C3adesARG response in subjects with familial combined hyperlipidemia [24]. Halkes et al. also showed an increase in plasma ASP/C3adesARG after oral lipid load in normolipemic subjects with coronary artery disease and normal volunteers [10]. Although this two studies showed an increase in C3adesARG in humans exposed to an oral lipid load, the results over the literature is not concordant. For instance, Charlesworth et al did not find any change to post-prandial plasma ASP/C3adesARG in healthy subjects submitted to an oral lipid load [9]. Even though, the authors observed an important individual variation in post-prandial plasma ASP/C3adesARG levels in their study. In our study, the changes in plasma ASP/C3adesARG also correlated to changes on triglycerides in control subjects during Intralipid^®^ and heparin infusion. The postprandial triglycerides clearance was delayed in MetS comparing to control group. In experimental study, Murray et al. already showed delayed post-prandial triglycerides clearance in ASP-deficient mice comparing to wild type [25]. ASP/C3adesARG is already known as a complement 3 fragment related to triglycerides clearance [8]. However, this is the first time that evidence of a blunted release of ASP/C3adesARG in subjects with the cluster of cardiovascular risk factors submitted to an Intralipid^®^ and heparin infusion, a model of acute hyperlipidemia, is showed. Since MetS group, which has higher fasting plasma triglycerides, did not show any increase in ASP/C3adesARG during Intralipid^®^ and heparin infusion and also showed lower triglycerides clearance after an Intralipid^®^ and heparin infusion, we can speculate a possible deficiency of ASP/C3adesARG synthesis in MetS subjects exposed to an acute hyperlipidemia.

## Conclusions

In conclusion, subjects with features of MetS have higher leptin, ASP/C3adesARG, and lower plasma level of adiponectin. They also showed lower triglycerides clearance and ASP/C3adesARG responses to an acute hyperlipidemia. These findings, taken together with previous reports, suggest that these adipocyte-derived factors play an important role in the pathogenesis and complications of MetS. The results further suggest that the levels of and balance between the various adipocyte derived factors may modulate the expression of various metabolic and hemodynamic facets of the syndrome. The differential impact of various adipocyte factors on individual components of the syndrome raises the potential for additional therapeutic targets.

## Abbreviations

MetS: Metabolic syndrome
ASP-C3ades/ARG: Acylation stimulating protein
BP: Blood pressure
BMI: Body mass index
NEFAs: Non-esterified fatty acids
FRAP: Ferric reducing activity of plasma
HOMAir: Homeostatic model assessment for insulin sensitivity
GCRC: General Clinical Research Center.
